# Gut microbiome and metabolic pathways linked to sleep quality

**DOI:** 10.3389/fmicb.2024.1418773

**Published:** 2024-07-31

**Authors:** Hoon Je Seong, Younghwa Baek, Siwoo Lee, Hee-Jeong Jin

**Affiliations:** Korean Medicine Data Division, Korea Institute of Oriental Medicine, Daejeon, Republic of Korea

**Keywords:** microbial composition, sleep quality, gut microbiome, *Faecalibacterium*, Pittsburgh Sleep Quality Index

## Abstract

Sleep quality is a vital determinant of human health as sleep disorders are associated with cognitive deficits, and chronic sleep deprivation is associated with a broad range of health complications. Previous studies on the association between the gut microbiome and sleep quality have been constrained by small sample sizes or have focused on specific sleep disorders, thus yielding inconsistent results. Herein, we investigated the relationship between microbial composition and sleep quality in a cohort of 159 Koreans. Sleep quality was measured using the Pittsburgh Sleep Quality Index (PSQI), determined through a self-administered questionnaire. Gut microbiome analyses were performed using 16S rRNA amplicons. We found no direct correlation between microbial alpha diversity metrics and sleep; however, we identified differences in beta diversity among sleep quality groups (with a PSQI score > 5 indicating poor sleep quality and PSQI ≤5 indicating good sleep quality). We also found differential microbial signatures (*Bacteroides*, *Prevotella* 9, and *Faecalibacterium*) among the groups. Furthermore, functional metabolic pathway profiles revealed significant linear correlations of the L-arginine and L-tryptophan biosynthetic pathways as well as 4-aminobutanoate degradation with sleep status. In particular, *Faecalibacterium prausnitzii*, which harbors these metabolic pathways, showed differences between sleep quality groups and a linear association with sleep quality scores and was thus identified as the species most strongly associated with sleep status. This study provides a significant advance in our understanding of the relationship between gut microbiota and sleep regulation. The current findings provide a basis for further research into potential therapeutic strategies for sleep disorders targeting the gut microbiome.

## 1 Introduction

Sleep quality is a vital determinant of human health and wellbeing. In recent years, the prevalence of sleep disorders has increased owing to lifestyle changes with the advent of telecommuting (Cabré-Riera et al., 2019), development of social media (Alonzo et al., 2021), and emergence of COVID-19 (Wehbe et al., 2022). Sleep disorders are intrinsically associated with cognitive deficits, and chronic sleep deprivation is associated with a broad range of health complications, including an elevated risk of inflammatory, metabolic, cardiovascular, neurological, and psychiatric diseases.

Sleep disorders are common and affect approximately 40–50% of the global population (Ford et al., 2014; Bin Heyat et al., 2021). Improving sleep quality remains a significant challenge owing to the severe adverse effects associated with Z-drugs (zolpidem, zopiclone, eszopiclone, and zaleplon), which can include cognitive impairment, falls, and amnesia (Gunja, 2013; Brandt and Leong, 2017), as well as with dopaminergic agents, known to cause and exacerbate the symptoms of sleep disturbance (Videnovic and Golombek, 2013; Ondo, 2014). Furthermore, the therapeutic options for addressing obstructive sleep apnea, such as surgery or positive airway pressure, are often inaccessible or inconvenient (Knauert et al., 2015). Given the pervasive impact of sleep disorders on millions of people worldwide and their severe effects on cognitive and physical function, the need to elucidate the relationship between sleep and various biological processes has emerged as a priority. Significant advances in our understanding of sleep biology have been made through large-scale genetic association studies (Jones et al., 2019; Campos et al., 2020; Sonti and Grant, 2022) and more recently, through comprehensive omics data analysis (Lahtinen et al., 2021; Wang et al., 2021).

The gut microbiome is emerging as an essential determinant of human health, associated with the occurrence of several metabolic and disease processes and the gut–brain axis (Zheng et al., 2021; Xiao et al., 2022; Zhou et al., 2023). Through this axis, the gut microbiome influences mood (Kuo and Chung, 2019), depression (Zheng et al., 2021), and psychiatric disorders, such as autism spectrum disorders (Mulle et al., 2013). Moreover, the microbiome undergoes changes in response to circadian rhythms (Tahara et al., 2018), and circadian disruption can affect gut microbiome homeostasis (Yang et al., 2023). Gut dysbiosis can lead to a decreased abundance of beneficial bacteria and exacerbation of inflammation, which can negatively impact the gut–brain axis and consequently affect sleep quality (Sen et al., 2021; Wang et al., 2021). Several studies have provided preliminary evidence for the involvement of the gut microbiome in sleep disorders in both mouse models and patients (Grosicki et al., 2020; Wang et al., 2021; Yang et al., 2023). These findings suggest that perturbations in sleep status can cause structural and functional changes in the gut microbiome, highlighting the close link between the gut microbiome and sleep.

Recently, the role of microbial-derived metabolites, such as short-chain fatty acids (SCFAs), serotonin, and melatonin, in sleep has garnered considerable attention. SCFAs, as the end products of microbial fermentation, are key signaling molecules that mediate communication between the gut microbiota and the host (Dalile et al., 2019). Butyrate, a predominant SCFA, has neuroprotective effects and is involved in the regulation of the circadian rhythm (Tahara et al., 2018; Dalile et al., 2019). Serotonin is primarily produced in the gut and is a crucial neurotransmitter that modulates mood and sleep (Bohórquez and Liddle, 2015). It is also a precursor of melatonin, a hormone synthesized and released by the pineal gland that is essential for sleep regulation (Jagota and Reddy, 2007). The gut microbiota is known to directly or indirectly influence the production of these neuroactive compounds (Yano et al., 2015; Dalile et al., 2019), thus potentially influencing the gut–brain axis and sleep (Magzal et al., 2021; Tang et al., 2022). These findings highlight the need to study the gut microbiome for gaining a deeper understanding into the pathophysiology of sleep disorders and potential treatment alternatives. However, to date, gut microbiome studies in relation to sleep quality in humans have been limited by sample size and inconsistent methods for assessing sleep status (Jackson et al., 2015; Grosicki et al., 2020; Agrawal et al., 2021), thereby making it challenging to estimate the impact of sleep.

Therefore, in this study, we aimed to investigate the characteristics of bacteria and their functional metabolic pathways associated with sleep quality. To interpret our results in terms of general sleep, regardless of a specific disease, we performed gut microbiome analyses on 159 healthy Korean participants using 16S rRNA amplicons. Based on the substantial number of participants and sleep quality evaluation, we analyzed alterations in the bacterial community structure via 16S rRNA sequencing. This allowed us to identify bacteria associated with sleep quality and characterize their functional metabolic pathways. The current findings shed light on the complex relationship between sleep and the gut microbiome in healthy individuals, offering general insights into microbiome-based interventions against sleep disorders.

## 2 Methods

### 2.1 Participants

To investigate the microbiome alterations associated with sleep quality, we recruited participants from the Korean Medicine Daejeon Citizen Cohort (KDCC) study (Baek et al., 2020), an ongoing cohort study to assess the relationship between lifestyle factors and chronic diseases. Exclusion criteria were as follows: antibiotic use for last 2 weeks; any cancer; any medical disability that interfered with a subject’s ability to complete study procedures. We did not exclude persons with metabolic diseases such as obesity, hypertension, and diabetes. Although these diseases may influence the stability of the gut microbiota, subjects with poor sleep quality tend to have higher rates of these metabolic medical conditions (Xia et al., 2021; Duan et al., 2023).

A total of 159 individuals (Supplementary Figure S1) consented to the provision of microbiome data from March to November 2023, and the study was conducted after they signed a consent form. We provided instructions for use and return, one sheet of waste collection paper, one tube for waste collection, one wet tissue for the bidet, and one return envelope to each subject. Samples were immediately discarded after analysis at the research institution. The Institutional Review Board of the Korea Institute of Oriental Medicine and the Regional Ethics Board of Dunsan Korean Medicine Hospital of Daejeon University reviewed and approved this study (IRB No. DJDSKH-17-BM-12). Written informed consent was obtained from all the participants.

### 2.2 Data collection

Sleep quality was measured based on the respondents’ subjective responses to the Korean version of the PSQI (Lee et al., 2020). The PSQI is a widely-used tool for assessing global sleep quality (Mollayeva et al., 2016). It assesses sleep latency, duration, habitual efficiency, disturbances, daytime sleepiness, sleep quality, and medication use (Buysse et al., 1989). The global PSQI score, which ranges from 0 to 21, can be calculated by summing the scores of the seven components on a scale ranging from 0 to 3, with higher scores indicating worse sleep quality. The PSQI is a reliable and valid tool for assessing sleep quality in clinical practice and research, and a global PSQI score of 5 or higher indicates poor sleep.

We used additional metadata on age, sex, blood pressure medication, and clinical information collected from the KDCC cohort to control for confounding factors that could affect the relationship between the microbiome and sleep. Specifically, the clinical information for the participants included the following five risk factors for metabolic syndrome (MetS) (Expert Panel on Detection, 2001): (1) systolic and diastolic blood pressure, (2) glucose level, (3) high-density lipoprotein (HDL) cholesterol level, (4) triglyceride level, and (5) waist circumference. We defined a MetS score when five risk factors crossed the cut-off according to gender-adjusted Korean-specific diagnostic criteria (Lee et al., 2007): (1) high blood pressure (systolic blood pressure ≥ 130 mmHg, diastolic blood pressure ≥ 85 mmHg) or specific treatment; (2) fasting plasma glucose ≥100 mg/dL or specific treatment; (3) low HDL cholesterol (<40 mg/dL for men, <50 mg/dL for women) or specific treatment; (4) high triglyceride levels (150 mg/dL) or specific treatment; and (5) abdominal obesity with cutoffs specific to South Koreans (waist circumference ≥ 90 cm for men or ≥ 85 cm for women).

### 2.3 Sample collection and DNA extraction

Stool samples were collected by participants in a hat that sits on the toilet seat. DNA was extracted from the fecal samples using a ARA MagNA Plant DNA Isolation Kit (LAS, Gimpo, Korea). Each fecal sample (200 μL) was transferred to a 2-ml tube containing 20 μL (40 mg/mL) proteinase k and 0.3 mL PL1 lysis buffer (LAS, Gimpo, Korea). After rotation with shaking for 10 min, samples were centrifuged to pellet the debris and lysing matrix at room temperature at 12,000 *g* for 2 min. After centrifuging, 0.4 mL of the supernatant was mixed with 0.4 mL PB2 binding buffer (LAS, Gimpo, Korea) and 20 μL magnetic beads. The subsequent DNA extraction steps were performed according to the manufacturer’s instructions. Eluted DNA was quantified with the Dropsense96. (PerkinElmer, Shelton, CT, United States) and stored at −20°C until use.

### 2.4 16S rRNA amplicon sequencing

A 16S rRNA amplicon sequencing library targeting the V3-V4 hypervariable region was prepared according to the Illumina 16S metagenomic sequencing library preparation protocol (Illumina, San Diego, CA, United States). Primary PCR used KAPA HiFi HotStart ReadyMix (KAPA Biosystems, Wilmington, MA, United States), with 10 ng of template DNA and V3-V4 specific primers (341F-805R), including Illumina sequencing indexes and adapters, were used. The primer sequences were as follows: 5′-TCGTCGGCAGCGTCAGATGTGTATAAGAGACAGCCTACGGGNGGCWGCAG-3′ (forward) and 5′-GTCTCGTGGGCTCGGAGATGTGTATAAGAGACAGGACTACHVGGGTATCTAATCC-3′ (reverse). The PCR products were purified using the Agencourt AMPure XP system (Beckman Coulter Genomics, Brea, CA, United States) and subjected to a limited-cycle amplification step with the Nextera XT Index Kit (Illumina). After a second purification, products were visualized via gel electrophoresis and quantified with a Qubit 3.0 fluorometer using the Qubit dsDNA HS Assay Kit (Thermo Scientific). The pooled libraries were assessed on an Agilent 2100 Bioanalyzer (Agilent Technologies) for size verification and quantification (CFX96 Real Time System [Bio-Rad]) prior to sequencing. Sequencing was performed on the Illumina MiSeq system with 300-bp paired-end reads.

### 2.5 Microbial community analysis

The microbial community was analyzed at the amplicon sequence variant (ASV) level using the Ampliseq pipeline (v2.4.1) (Straub et al., 2020) based on the DADA2 algorithm. AmpliSeq consists of a series of analyses in the following programs using the NextFlow pipeline: FastQC (v0.11.9), Cutadapt (v3.4), DADA2 (v.1.22.0), and QIIME2 (v2019.10.0). The taxonomy of ASV was assigned using the ‘assignTaxonomy’ module in DADA2 with SILVA database (v.138); however, we only utilized the results of the ‘assignSpecies’ module because species assignment requires a 100% exact match (Edgar, 2018).

Microbial diversity indices were calculated after rarefaction at the minimum sample depth (29,798 reads) for all the samples. Alpha diversity was calculated using evenness, Faith’s phylogenetic diversity (PD), observed features, and Shannon’s H index. Beta diversity measures the differences in ASV communities between samples using the Bray–Curtis and UniFrac indices. The association of the individuals’ clinical data with the Bray–Curtis distance matrix was calculated using the vegan’s envifit module.

### 2.6 Functional pathway prediction

PICRUSt2 (v2.5.0) (Douglas et al., 2020) was used to predict metagenomic functional pathways. PICRUSt2 is a software tool that predicts metagenomic functional attributes based on phylogenetic information from the ASV dataset. Briefly, we inferred the function of the ASVs generated by DADA2 based on a pre-constructed phylogenetic tree. The unstratified MetaCyc pathways were then converted into relative abundances for subsequent differential testing. We also used the “stratified” option to infer the ASVs assigned to each metabolic pathway.

### 2.7 Statistical analysis

Linear discriminant analysis (LDA) effect size (LEfSe) (Segata et al., 2011) was used to identify potential bacterial markers that could distinguish between the sleep quality groups. LEfSe first used between-group statistical tests (Kruskal-Wallis, *p* < 0.05) to detect features that were significantly different between the sleep quality groups. LDA was used to estimate the effect size of each differentially enriched component, which indicated biological relevance (LDA score > 2).

Microbial balance signatures discriminating sleep quality were identified using self-analysis. The optimal number of microbial balance variables was chosen using the Selbal (Rivera-Pinto et al., 2018). cv. module with 5-fold cross-validation. To avoid bias from rare ASVs, we used only ASV and species abundances that represented a 20% prevalence in all samples with at least 10 reads.

To adjust for clinical characteristics that may affect the microbiome, we used a multivariate association with linear models (MaAsLin2) (Mallick et al., 2021) algorithm to test the microbial taxonomy and functional metabolic pathways related to sleep quality scores. Microbial features that passed the significance threshold (adj. *p* false discovery rate [FDR] < 0.25) are shown in the MaAsLin2 analysis comparing global PSQI scores after adjusting for age, sex, MetS score, and blood pressure medication status.

### 2.8 Co-occurrence network analysis

To infer the gut microbiota co-occurrence networks of the two groups according to sleep quality, we used SPIEC-EASI (Kurtz et al., 2015) with ASV relative abundance data. Only ASVs with a total sample frequency of at least 20% and more than two read counts were selected to reduce bias from rarely detected ASVs during the co-occurrence analysis. The R package igraph was used to calculate the edge density (*D*) and network transitivity (*T*) values. We filtered out negative relationships between ASVs to calculate network modularity and used Cytoscape (Shannon et al., 2003) for subsequent network visualization.

## 3 Results

### 3.1 Microbial community structure according to sleep quality

In this study, we analyzed the microbial communities of 159 participants, of which 99 (62.26%) belonged to the good sleep quality (GS) group and 60 (37.74%) to the poor sleep quality (PS) group (PSQI score > 5), as detailed in Supplementary Table S1 (*p* > 0.05). No significant differences in the metadata characteristics were observed among the participants in either group. Additionally, no correlation was identified between the sleep quality metric (PSQI) and age, sex, or other metadata associated with MetS (Supplementary Figure S2, *p* > 0.05).

**Figure 1:**
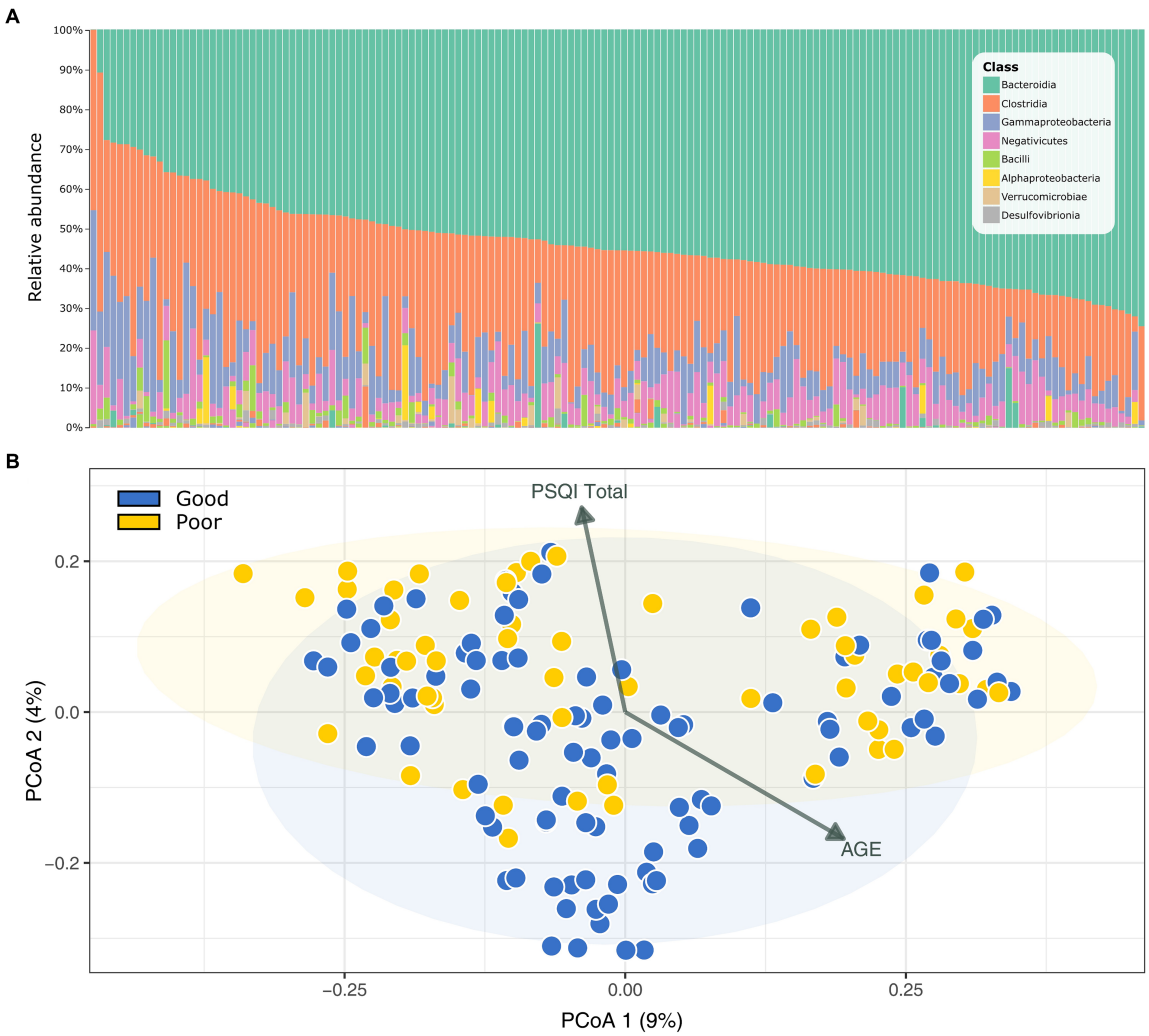
Gut microbial community profile of the Korean Medicine Daejeon Citizen Cohort. **(A)** Microbial community composition at the class level. **(B)** Principal coordinate analysis (PCoA) of Bray–Curtis dissimilarity in sleep quality groups (PERMANOVA, *p* = 0.004). PSQI, Pittsburgh Sleep Quality Index.

Our analysis of the gut microbial community using 16S rRNA amplicon sequencing yielded over 12.7 million quality-filtered reads (76,878 ± 31,623 sequences per sample). The DADA2 algorithm generated a final set of 7,528 ASVs for subsequent downstream analyses. This set included 13 bacterial phyla and two archaeal phyla, with *Bacteroidetes*, *Firmicutes*, and *Proteobacteria* being the most abundant and evenly distributed across all samples, regardless of sleep quality (Figure 1A).

When comparing the microbial diversity between the GS and PS groups using various indices (including α-diversity measures: observed ASVs, Shannon, Faith’s phylogenetic diversity; and β-diversity measures: Bray–Curtis and UniFrac distances), we observed no differences in α-diversity. However, a significant difference was noted in β-diversity (Bray–Curtis, PERMANOVA, *p* = 0.004), suggesting a variation in microbial community structure between the groups based on sleep quality (Figure 1B). We further analyzed the linear relationship of the principal coordinate analysis ordination with other metadata using the Envfit function (Figure 1B). The total PSQI score (*R*^2^ = 0.075, *p* = 0.0025) and age (*R*^2^ = 0.066, *p* = 0.0058) showed contrasting associations, indicating that while these metrics were not correlated with each other, each had a distinct and significant effect on the gut microbiome.

### 3.2 Differential microbiome signatures across sleep quality groups

**Figure 2:**
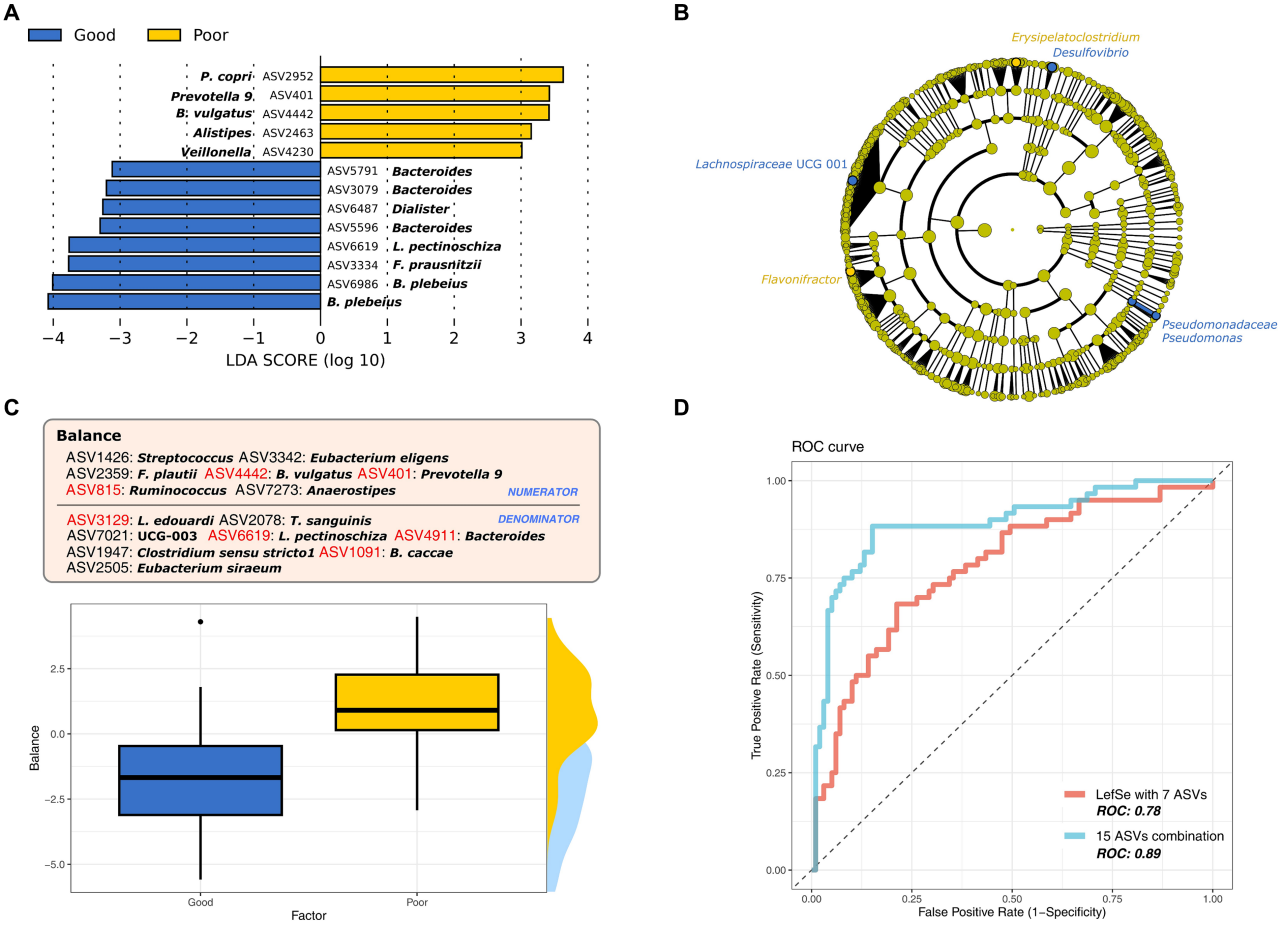
The most differential microbiome signatures according to sleep quality groups. **(A)** Major bacterial taxa according to sleep quality group based on the linear discriminant analysis (LDA) score > 3. **(B)** Cladogram of lineage specific markers based on LDA scores (>2) according to sleep quality. **(C)** The best microbial balance signature to discriminate sleep quality groups at the amplicon sequence variant (ASV) level. **(D)** ROC curve for sleep quality markers (ASVs) identified by LefSe and the balance signature.

To identify the biomarker taxa that showed significantly differential enrichment in the GS and PS groups, we used LEfSe analysis. Predominantly, these biomarkers reflected the ASV at the genus and species levels (LDA ≥ 3 and *p* < 0.05). *Bacteroides* was the most enriched genus in both groups, with unique differences at the ASV and species levels; *Bacteroides plebeius* was identified in the GS group, while *B. vulgaris* was found in the PS group (Figure 2A). Furthermore, significant ASVs of *Prevotella* group 9, *Alistipes*, *Alloprevotella*, and *Veillonella* were observed in the PS group, whereas the GS group was marked by ASVs of *Faecalibacterium prausnitzii*, *Lachnospira pectinoschiza*, *Bacteroides*, and *Dialister*. Reducing the LDA score threshold to 3 to 2 for both groups (*p* < 0.05) enabled the identification of previously undetected biomarkers at the genus level, such as *Erysipelatoclostridium*, *Flavonifractor*, *Desulfovibrio*, *Lachnospiraceae* UCG 001, and *Pseudomonas*. At the family level, *Pseudomonadaceae* was specific to the GS group (Figure 2B).

We expanded our investigation to explore microbial ratios, termed as “balance,” instead of solely focusing on individual taxon markers. This approach aims to uncover a more detailed microbial signature associated with sleep quality (Figure 2C). In line with our LEfSe analysis, balance confirmed that the ASV level provided the most accurate discrimination between the sleep groups (Figure 2D; cross-validated area under the curve [AUC]: 0.89), followed by the family (cross-validated AUC: 0.64) and genus (cross-validated AUC:0.74) levels. The balance consisted of 15 ASVs, with seven overlapping with prior LEfSe results (LDA ≥ 2), which also demonstrated significant discriminatory power for sleep quality (Figure 2D; cross-validated AUC: 0.78). The PS group was represented by the numerators (ASV401, ASV815, and ASV4442) and the GS group by the denominators (ASV1091, ASV3129, ASV4911, and ASV6619) of the balance. Additionally, we identified ASVs associated with *Anaerostipes*, *Clostridium*, *Eubacterium*, *Flavonifractor*, *Streptococcus*, and *Turicibacter*, which differed from the LEfSe findings. Their combined analysis allowed us to more precisely identify the microbial markers linked to sleep quality, suggesting that these findings are due to differences in microbial composition, further supported by the beta diversity results.

### 3.3 Co-occurrence networks in the microbial communities

**Figure 3:**
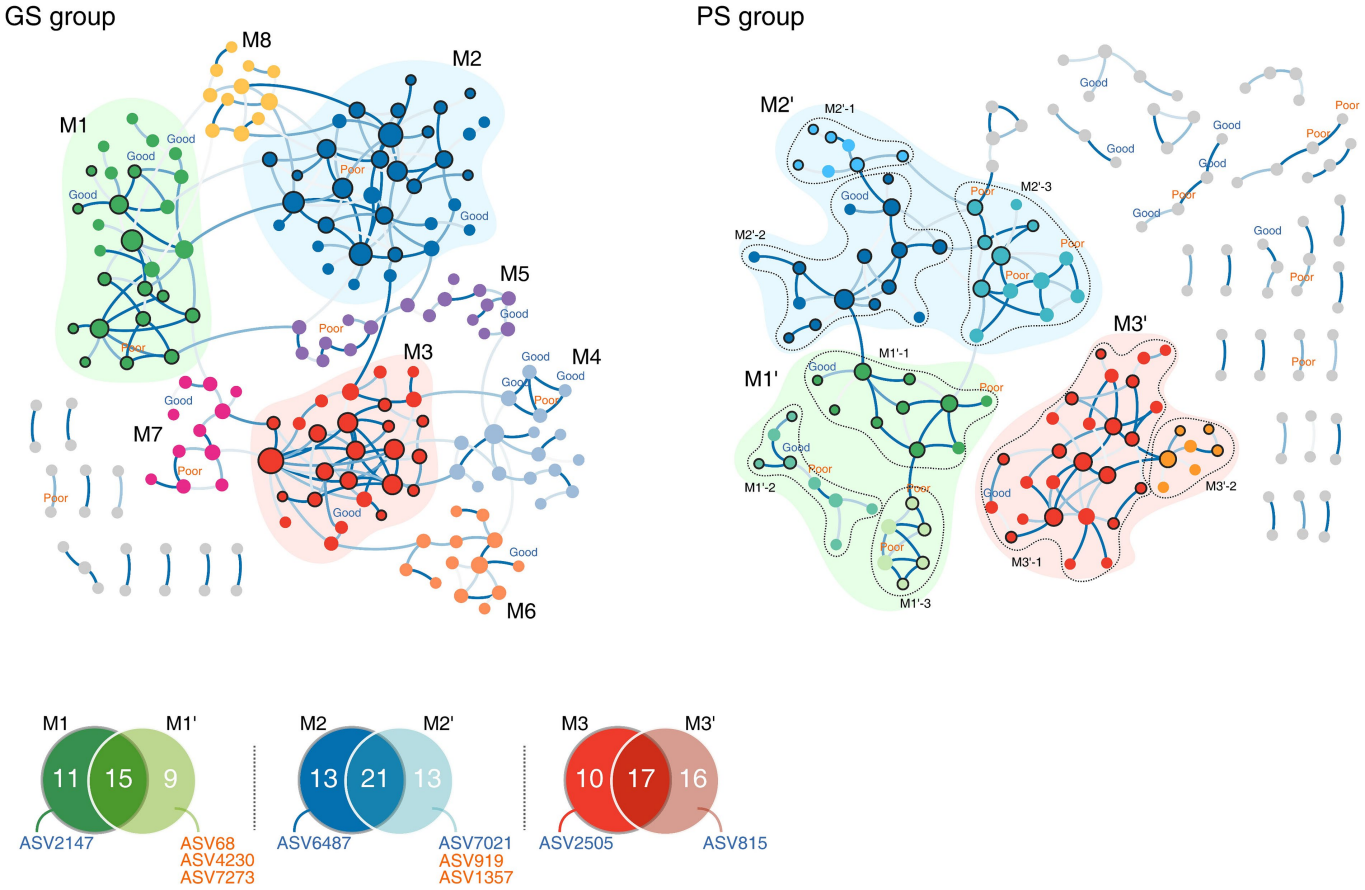
Microbial co-occurrence networks according to sleep quality groups. Nodes in bold are sleep quality group markers, shown with their corresponding amplicon sequence variant (ASV) IDs. GS, good sleep quality; PS, poor sleep quality.

To further explore the bacterial interactions relative to sleep quality groups, we constructed microbial networks. The GS and PS group networks consisted of 222 and 232 ASVs, respectively, with 197 ASVs shared across the networks. However, the network topology diverged between the two groups, with *D* and *T* (clustering coefficient) being higher in the GS group (*D*: 0.015, *T*: 0.147) than in the PS group (*D*: 0.008, *T*: 0.123). Although the overall network structure was similar between the two groups, the PS group exhibited fewer substantial interactions. Notably, the positive inter-microbial clusters that dominated the GS group were disorganized and diminished in the PS group, although they retained their form Figure 3.

Calculating modularity based on the connectivity of each ASV yielded eight modules for each sleep quality group. Among these, three modules in the GS group (M1–M3) exhibited a topology similar to the eight modules in the PS group (referred to as M1’–M3’, respectively), sharing numerous ASVs belonging to the *Lachnospiraceae* and *Oscillospiraceae* families. Despite 42.4% of all modules sharing ASVs, the marker ASVs for each group remained unique to that group (Figure 3). In the GS group, modules M1 and M2 were split into three modules each in the PS group (labeled as M1’ 1–3 and M2’ 1–3) with the addition of PS group-specific marker ASVs not found in the GS group (Figure 3). Notably, in the PS group, marker ASV2359, along with two other PS group marker ASVs (ASV919 and ASV1357) associated with *Erysipelatoclostridiaceae*, formed the PS group-specific module M2’-3. Conversely, five modules identified in the GS group (M4–M8) were found to be de-modularized in the PS group, highlighting the stable nature of bacterial interactions within the GS group and suggesting a more consistent co-occurrence pattern. While the microbial network backbones of the two groups were similar, they differed in their specific microbial interactions, which in turn affected the stability of each group’s microbial community.

### 3.4 Linear associations between metabolic pathway profiles and PSQI scores

Next, we aimed to understand the link between sleep quality and the microbiome by directly associating PSQI scores with the microbiome rather than dividing samples based on sleep quality. After adjusting for age, sex, MetS score, and blood pressure medication, we examined both individual microbial taxa and metabolic pathways.

**Figure 4:**
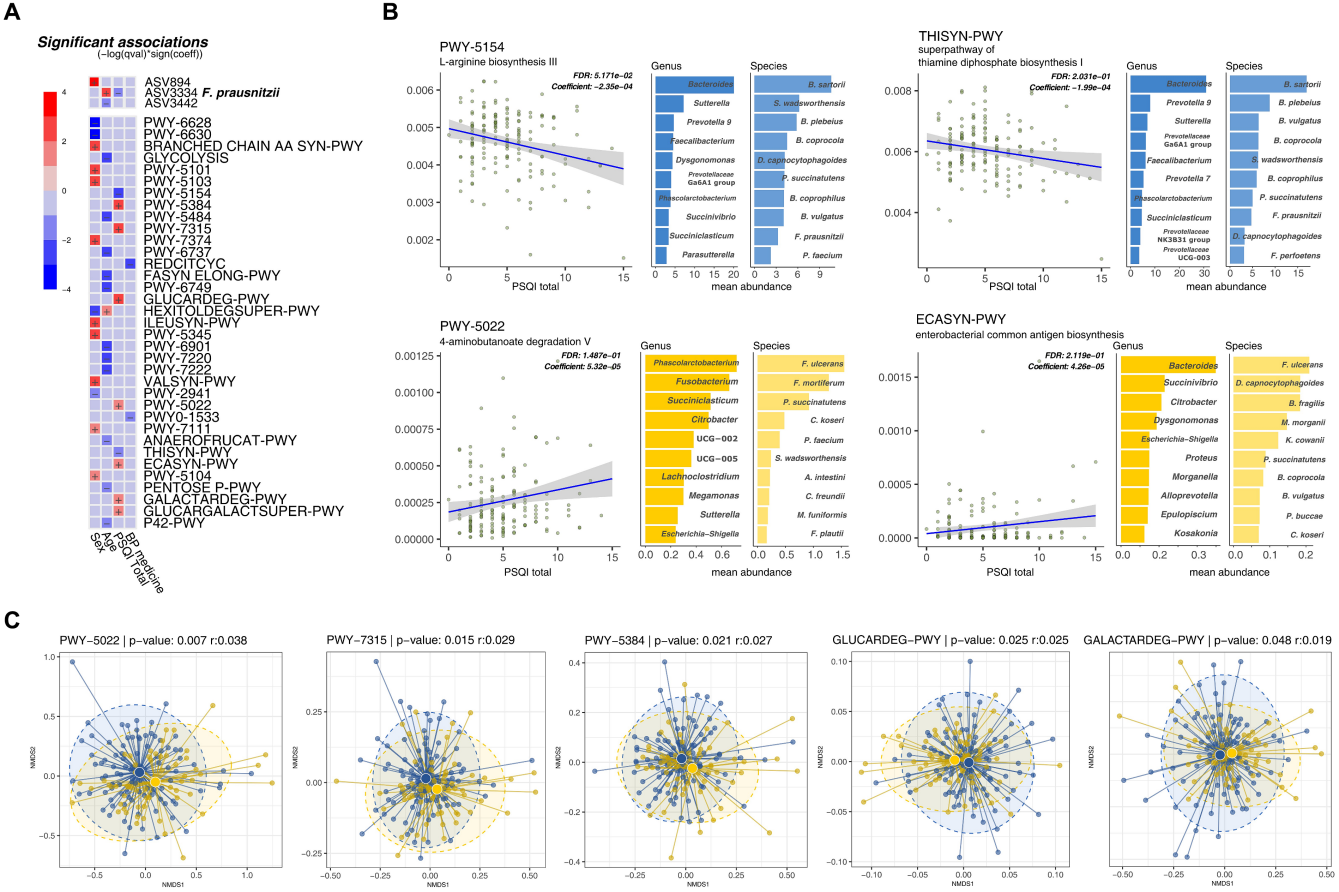
Metabolic pathways inferred by PICRUSt2 correlate with sleep quality scores (global Pittsburgh Sleep Quality Index [PSQI]). **(A)** Multivariate association test of metabolic pathways with PSQI and other clinical metadata. **(B)** Significant metabolic pathways with a linear relationship with sleep quality scores. The mean metabolic pathway abundance of the top 10 bacterial species with each metabolic pathway. **(C)** NMDS analysis of taxonomic stratified metabolic pathway.

Among the microbial taxa, only ASV3334 (*F. prausnitzii*) showed a negative correlation with the PSQI scores (adjusted *p* = 0.23). However, within the metabolic pathway profiles, several significant metabolic pathways were identified that associated with sleep quality independently of other metadata (including age, sex, MetS score, and blood pressure medication). Specifically, seven factors showed a positive correlation with PSQI scores, while two factors exhibited a negative correlation (adjusted *p* < 0.25) (Figure 4A).

Most degradation pathways, including sucrose, dicarboxylic acid sugars (D-glucarate and galactarate), and 4-aminobutanoate (GABA), were positively correlated with PSQI scores (PWY-5384, GLUCARDEG-PWY, GALACTARDEG-PWY, GLUCARCALACTSUPER-PWY, and PWY-5022). Pathways related to the synthesis of common *Enterobacteriaceae*-associated antigens (PWY-7315 and ECASYN-PWY) also showed positive correlations with the PSQI scores. In contrast, biosynthetic pathways for the production of amino acids, including L-arginine (PWY-5154) and thiamine diphosphate (THISYN-PWY), were negatively correlated with the PSQI scores (Figure 4B).

Nine metabolic pathways demonstrated substantial differences in taxonomic richness based on their association with PSQI scores. The two pathways negatively associated with PSQI scores corresponded to an average of 202.5 genera. Conversely, the seven pathways positively correlated with PSQI scores were linked to an average of 69.14 genera, indicating significant disparities in taxonomic diversity of functional significance. Moreover, when comparing the microbial communities of these metabolic pathways using nonmetric multidimensional scaling plots, the ASV composition showed significant differences between the sleep groups, indicating that the diversity of microorganisms within the metabolic pathways varied (Figure 4C). Beyond these diversity differences, two pathways associated with good sleep quality (negatively correlated with PSQI scores) were predominantly enriched in two species previously found to be markers of the GS group: *B. plebeius* and *F. prausnitzii* (Figure 4B). These results indicate that differences in metabolites resulting from changes in the microbiome are associated with an individual’s sleep quality.

## 4 Discussion

A growing body of evidence suggests that the microbiota–gut–brain axis plays an integral role in the etiology and progression of sleep disorders by directly or indirectly contributing to sleep regulation. Disturbances in sleep patterns have been linked to changes in gut microbiota composition, whereas sleep deprivation has been linked to gut microbiota dysfunction. However, existing research has mainly focused on individual sleep disorders, such as apnea or insomnia, with small sample sizes (Han et al., 2022) often representing a limitation. Consequently, a comprehensive understanding of the association between sleep quality and the microbiome is warranted.

In this study, we explored the potential links between the microbiome, its functional pathways, and sleep quality in a cohort of 159 individuals from South Korea. Although our findings did not indicate any significant correlation between microbial diversity metrics and MetS within the sampled cohort (Supplementary Figure S2, Supplementary Table S1), we noted substantial differences in beta diversity (Bray–Curtis dissimilarity) and microbial signatures that could be used to distinguish between groups based on sleep quality. Moreover, observing a more stabilized microbiome in the GS group underscores the close interplay between sleep quality and the gut microbial community.

Here, we observed several microbial markers, such as *Alistipes*, *Bacteroides*, *Dialister*, *Faecalibacterium*, and *Veillonella*, that showed significant differences between the sleep quality groups. This is in line with previous findings linking these microbes with the gut–brain axis and various sleep and psychiatric disorders. For example, *Bacteroides*, and *Veillonella* are often overrepresented in major depressive disorders, whereas *Faecalibacterium* and *Dialister* are underrepresented (Kuo and Chung, 2019). *Alistipes* is also enriched in major depressive disorders (Zhang et al., 2021), possibly regulating emotions through indole metabolism (Song et al., 2006). In sleep-related studies, the abundance of *Faecalibacterium* and *Prevotella* group 9 decreased in a chronic insomnia group (Li et al., 2020) and that of *Alloprevotella* and *Prevotella* decreased with declining fecal SCFA concentrations after sleep deprivation (Wang et al., 2021). However, *Prevotella* was identified as a marker for the PS group in this study. When considered alongside previous findings (Grosicki et al., 2020), which reported that *Prevotella* was positively correlated with the PSQI score, the results appear inconsistent. Furthermore, studies on sleep disorders due to acute sleep deprivation (Li et al., 2020) and traumatic brain injury (Zhanfeng et al., 2022) have reported an increase in the abundance of *Bacteroides* upon sleep disturbance. However, our findings indicate that at the ASV and species level, *B. plebeius* and *B. vulgatus* exhibit opposite patterns between the sleep groups, suggesting a need for more detailed analyses beyond the genus level. This apparent contradiction could be because of differences in study design, cohort characteristics, or unaccounted environmental factors. Such discrepancies underscore the need for further research to resolve inconsistent conclusions in sleep research regarding specific changes in bacterial composition (Reutrakul et al., 2020; Morwani-Mangnani et al., 2022).

The SCFA butyrate can be directly transmitted via the vagus nerve, acting as a signaling molecule to induce sleep onset (Szentirmai et al., 2019). However, this mechanism is drastically altered under specific pathophysiological conditions (Magzal et al., 2021). Although SCFAs have been shown to inhibit inflammation and promote sleep in several observational studies (Szentirmai et al., 2019), paradoxically, a recent study reported that low sleep efficiency in older adults corresponds to higher fecal SCFA levels (Magzal et al., 2021). Consequently, SCFAs may influence sleep by modulating inflammation and the gut–brain axis; however, the precise metabolic links remain ambiguous and require further investigation (Magzal et al., 2021; Morwani-Mangnani et al., 2022). In this study, marker microbes (*Bacteroides*, *Prevotella*, *Veillonella*, *L. pectinoschiza*, and *F. prausnitzii*) were strongly associated with SCFA production (Ng and Hamilton, 1973; Schwiertz et al., 2010; Zhang et al., 2015; Koh et al., 2016; Liu et al., 2018; Cheng et al., 2022), suggesting a possible link between sleep quality and bacterial composition. However, these markers were found in both the GS and PS groups regardless of sleep quality. Furthermore, one limitation of the present study is that SCFAs were not directly detected. Our results, in addition to those of previous studies, do not provide sufficient evidence to conclude that SCFAs directly affect sleep quality.

Building on the observations of microbial markers, we further examined the functional metabolic pathway profiles to ascertain potential associations with sleep quality. In this regard, we found correlations between the PSQI and certain pathway markers. Specifically, the biosynthetic pathways of L-arginine and thiamine diphosphate (vitamin B1 derivative) positively correlated with better sleep quality (negatively correlated with the PSQI score) and were closely related to sleep induction. L-arginine, a precursor to the neurotransmitter nitric oxide, is known to induce sleep (Gautier-Sauvigné et al., 2005) and augment slow-wave sleep (Monti and Jantos, 2004). Supplementation with L-arginine has been shown to ameliorate pathological changes caused by REM sleep deprivation (Jiang et al., 2017). Additionally, vitamin B1 plays a pivotal role as a cofactor in glucose metabolism and is deeply involved in the regulation of the activity of the central nervous system, acting as a primary source for the metabolism of neurotransmitters such as glutamate, GABA, and acetylcholine (Rudzki et al., 2021). Deficiencies in vitamin B1 have been linked to various psychiatric conditions, including sleep disturbances (Dhir et al., 2019).

Furthermore, the L-tryptophan biosynthetic pathway was enriched in the GS group (adj. *p* FDR = 0.15); the enrichment of this pathway was suppressed, according to the PSQI score (*p* = 0.01, adj. *p* FDR = 0.3). L-tryptophan is the sole precursor of serotonin (Richard et al., 2009) and exerts a direct influence on the gut–brain axis, mood, and sleep (Kikuchi et al., 2021), with reports linking L-tryptophan supplementation to a decreased degree of depression, increased sleep duration (Lieberman et al., 2016), and amelioration of sleep disorders (Silber and Schmitt, 2010). Remarkably, the therapeutic effects of tryptophan in sleep disorder treatment are mediated via melatonin, without impairing cognitive performance or suppressing wakefulness during sleep (Richard et al., 2009).

In contrast, the microbial pathways involved in the degradation of GABA (PWY-5022) were negatively correlated with improved sleep quality. GABA, the most common inhibitory neurotransmitter, is associated with the enhancement of hyperarousal in insomnia (Riemann et al., 2010; Plante et al., 2012) when its levels are decreased and has been associated with shorter sleep duration (Spiegelhalder et al., 2016). Furthermore, SCFAs are the products of GABA degradation, and the increased abundance of SCFA-producing microbes in the PS group may thus be related to the enhancement in the activity of GABA-degrading metabolic pathways. This finding reveals a potential link between sleep quality and metabolic pathways, such as the L-arginine, L-tryptophan, vitamin B1, and GABA metabolic pathways, further suggesting that they might influence the gut–brain axis more prominently than SCFAs.

*F. prausnitzii*, one of the most abundant bacteria in the gut, has attracted significant interest because of its potential role in gut health (Lopez-Siles et al., 2017). It is a key SCFA butyrate-producing bacterium in the gut and has been found to be negatively associated with various inflammatory bowel diseases, such as ulcerative colitis and Crohn’s disease; these findings suggest that this bacterium is a potential marker of gut health (Miquel et al., 2013). Although no studies have directly linked *F. prausnitzii* to sleep, it is one of the bacterial taxa whose abundance is reduced by circadian rhythm changes (Mortaş et al., 2020) and obstructive sleep apnea syndrome (Valentini et al., 2020). In our study, ASV3334, which belongs to *F. prausnitzii*, was the only microbial marker that was abundant in the GS group; its abundance increased linearly with sleep quality. This ASV is also predicted to involve metabolic pathways associated with sleep quality. As our study was conducted in a Korean cohort from Daejeon, South Korea, it may have contributed to the identification of a specific ASV rather than a species. Thus, our findings suggest a potential association between *F. prausnitzii* and sleep quality, extending insights from circadian rhythm studies (Mortaş et al., 2020). However, additional studies are required to validate this association in different ethnic populations with various lifestyles.

In conclusion, our study unveiled a potential association between the gut microbiota composition, their metabolic functions, and sleep quality, pinpointing key distinctions in microbial diversity among groups defined by sleep quality. However, as an observational cohort study, our study has certain limitations that affect the interpretation of microbial changes, such as diet history and food intake. In addition, we could not directly detect metabolite changes in blood or fecal samples or utilize controlled interventional designs to establish causality. Therefore, future *in vivo* studies on the sleep-related marker microbes and metabolomes introduced in this research are necessary in order to uncover the mechanisms underlying the gut microbiota-mediated enhancement of sleep quality. Overall, this study broadens our understanding of the complex interplay between sleep quality and the microbiome, providing a basis for future therapeutic strategies. These strategies could help in alleviating sleep disorders by modifying the microbiome with probiotics and prebiotics, and future causality studies aimed at influencing the markers identified in the present study will be conducted.

## Data availability statement

The raw sequence data have been archived at the NCBI Sequence Read Archive under accession number SRR28557588 ~ SRR28557746 and BioProject accession number PRJNA1096121.

## Ethics statement

The studies involving humans were approved by the Institutional Review Board of the Korea Institute of Oriental Medicine and the Regional Ethics Board of Dunsan Korean Medicine Hospital of Daejeon University reviewed and approved this study (IRB No. DJDSKH-17-BM-12). The studies were conducted in accordance with the local legislation and institutional requirements. The participants provided their written informed consent to participate in this study.

## Author contributions

HS: Conceptualization, Methodology, Visualization, Writing – original draft, Writing – review & editing. YB: Data curation, Writing – review & editing. SL: Data curation, Writing – review & editing, Funding acquisition. H-JJ: Conceptualization, Writing – original draft, Writing – review & editing.
