# Supplementary material for: Gut microbiome and metabolic pathways linked to sleep quality
Source: Front Microbiol. 2024 Jul 31;15:1418773. doi: 10.3389/fmicb.2024.1418773 (PMC11322573; doi:10.3389/fmicb.2024.1418773)
Supplement: Supplementary file 2 [file Data_Sheet_1.DOCX]

Supplementary Material

# Supplementary Figures


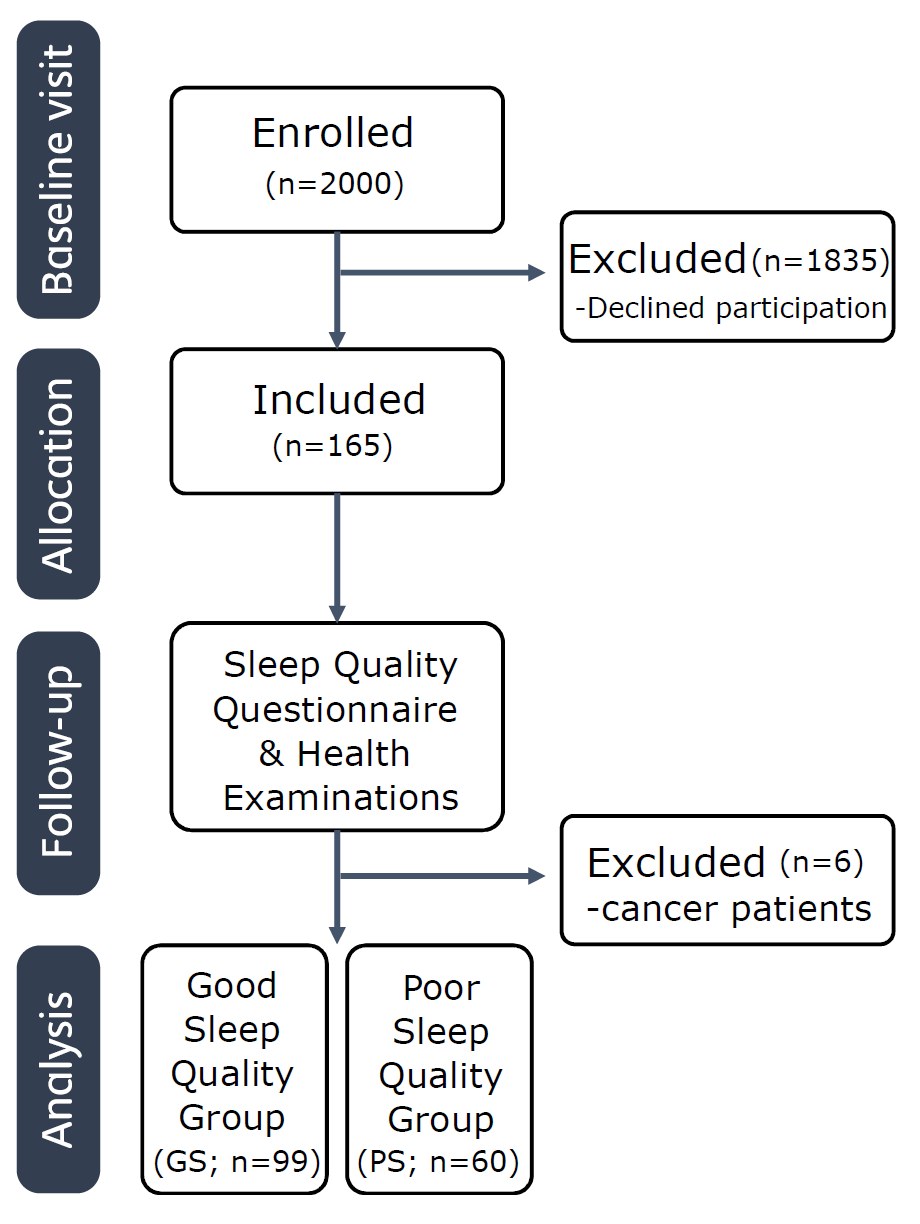


**Supplementary Figure 1.** Cohort study flow diagram.


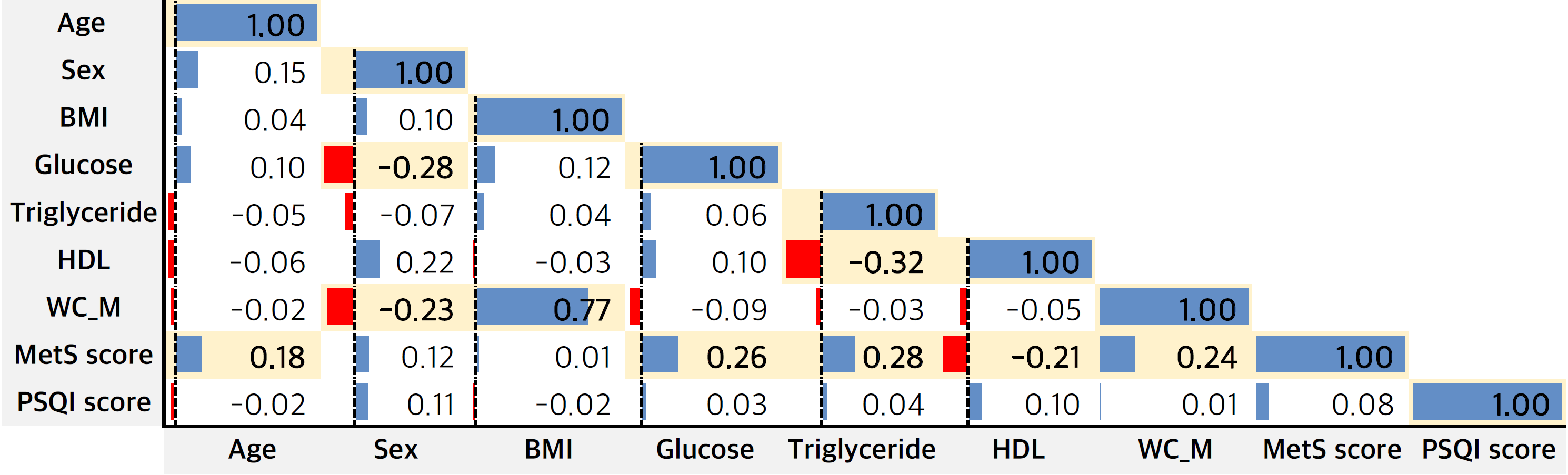


**Supplementary Figure 2.** Spearman correlation between clinical metadata. Estimated values are shown, with significant values (*p* <0.05) in bold. BMI, body mass index; HDL, high-density lipoprotein; MetS, metabolic syndrome; PSQI, Pittsburgh Sleep Quality Index; WC_M, mean of waist circumference
